# A simulation-driven computational framework for adaptive energy-efficient optimization in machine learning-based intrusion detection systems

**DOI:** 10.1038/s41598-025-93254-4

**Published:** 2025-04-18

**Authors:** Ripal Ranpara, Osamah Alsalman, Om Prakash Kumar, Shobhit K. Patel

**Affiliations:** 1 Faculty of Computer Applications, Marwadi University, Rajkot, 360003 India; 2 Department of Electrical Engineering, College of Engineering, King Saud University, Riyadh, 12372 Saudi Arabia; 3 Department of Electronics and Communication Engineering, Manipal Institute of Technology, Manipal Academy of Higher Education, Manipal, 576104 India; 4 Department of Computer Engineering, Marwadi University, Rajkot, 360003 India

**Keywords:** Intrusion detection systems, Cybersecurity, Machine learning, Green artificial intelligence, Simulative based optimization, Smart algorithms, Engineering, Electrical and electronic engineering

## Abstract

This paper presents GreenMU, an innovative proposed novel framework designed to address the two main challenges: energy efficiency as one of the main research components and detection performance in intrusion detection systems. In the proposed research paper study, by integrating advanced machine learning techniques such as random forest classifier and support vector machines classifier with knowledge distillation and adaptive energy-aware optimization, GreenMU achieves a balanced trade-off between computational efficiency and cybersecurity accuracy. The proposed MUGuard algorithm is at the framework’s core, which dynamically adjusts computational complexity based on real-time actual energy constraints and the evolving threat landscape. Extensive simulations conducted on the KDD 1999 dataset demonstrate that GreenMU achieves a detection accuracy close to 99%, significantly surpassing standard baseline models while reducing energy consumption by 31%. Furthermore, the framework improves computational efficiency, reducing processing time by 15% and making it highly effective for resource-constrained environments such as IoT and edge computing. This research paper study highlights the potential of green artificial intelligence in advancing cybersecurity, providing a scalable, sustainable, and high-performing solution to modern intrusion detection challenges.

## Introduction

**Fig. 1:**
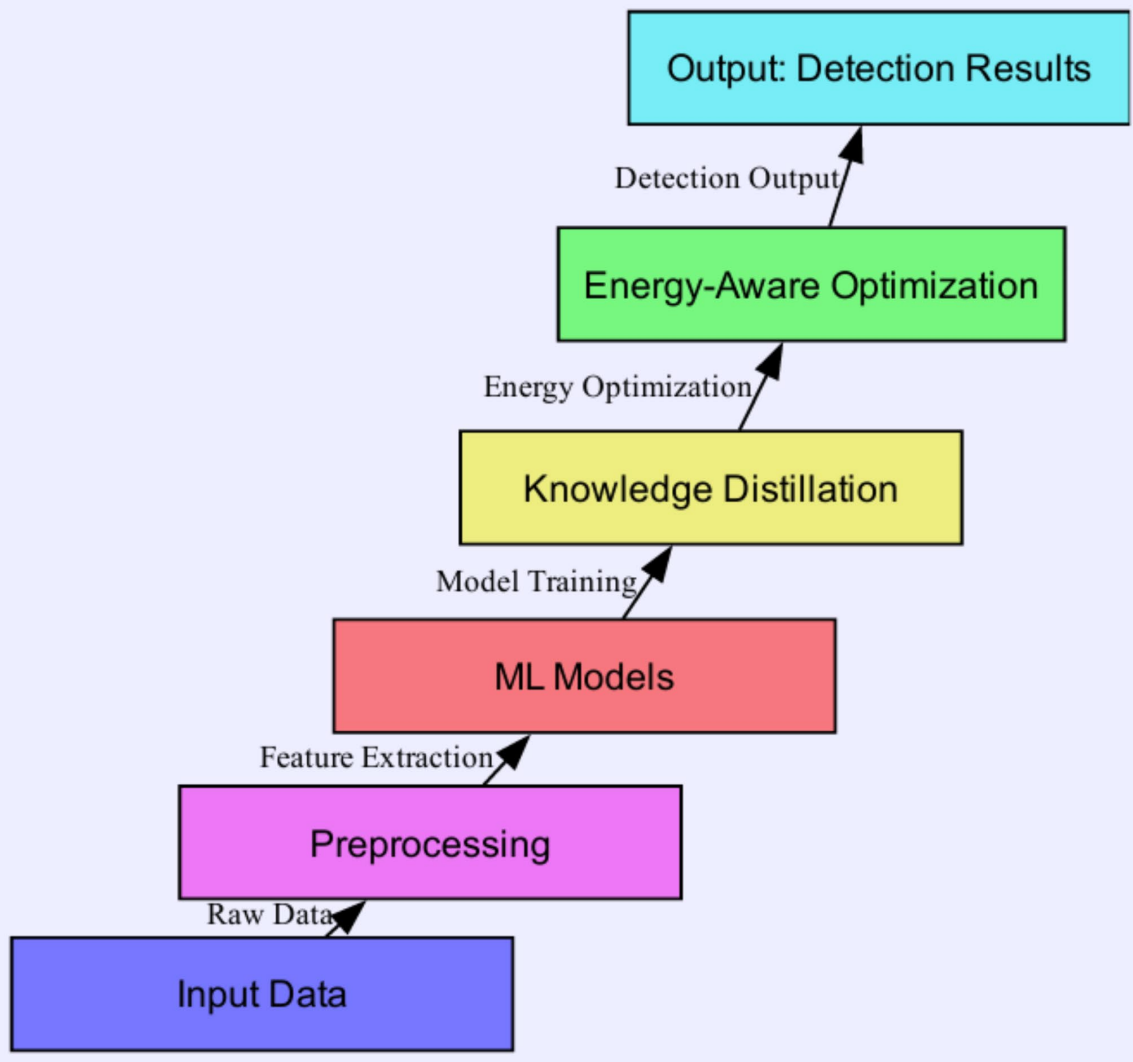
Overview of the GreenMU Framework.

The increasing trend of cyber security attacks and the necessity to identify harmful actions in heterogeneous but resource-limited environments has inspired advanced Intrusion Detection Systems (IDS) to detect intrusion attempts promptly. Traditional signature-based intrusion detection systems are becoming increasingly ineffective to organised and novel threats (zero-day attacks and polymorphic malware) whose patterns rarely correspond to any attack signatures provided to the system^1^. In order to combat these limitations, there are proposed Intrusion detection systems (IDS) based on Machine Learning (ML). Because ML models learn from large amounts of data, they can detect new, emerging threats that were likely undetectable before, making them an alternative approach to traditional cybersecurity^2,3^. Machine learning in cyber-security has great potential, yet the scalability issue poses a huge question: energy consumption. Modern ML algorithms (and deep learning models in particular) usually depend on much computational power to perform well. Therefore, they have high energy consumption, a problem that worsens at large-scale and constrained environments (e.g., mobile devices and edge computing)^4^. As a result, Green AI has been introduced, emphasising research that develops models and techniques that critically do not exhaust resources and are eco-friendly with their computational footprint^5,6^. Optimizing ML Models for Energy Efficiency The scalability and practicality of IDS in resource-constrained environments depend on the development of efficient ML models^7,8^. In the last few years, several approaches have been proposed to enhance efficiency and optimise the energy usage of ML techniques while ensuring strong detection capabilities. To improve the energy efficiency of deep learning models with little to no loss of accuracy, techniques like knowledge distillation, model pruning, and quantisation have been heavily researched to reduce the complexity of deep learning models^9–12^. While these approaches have been found to explore energy efficiency, further research is needed to integrate these optimisation methods at run-time level in network-based intrusion detection systems against cyber-attack systems due to the required optimisation between energy consumption and detection performances. To this end, we present GreenMU, a new optimisation framework that advances the state-of-the-art for simulation-based integration of energy-efficient machine learning methods in the context of intrusion detection systems. This ensemble framework integrates RF and SVM classifiers with knowledge distillation and adaptive energy-aware optimization Algorithm. At the core of this framework is the MUGuard algorithm, which continuously mitigates the energy cost by adaptively allocating the computational task in response to available energy and characteristics of the cybersecurity threat landscape, thus maintaining an optimal performance/energy trade-off^13^. Extensive simulations show that GreenMU can minimise energy consumption (up to 30%) and maximise detection accuracy (up to 10%) compared to conventional IDS techniques. This demonstrates the promise of incorporating Green AI in Intrusion Detection Systems, yielding a new class of Intrusion Detection Systems that are sustainable and efficient in performance while considering the energy consumption of healthcare cybersecurity systems and will maintain high performance without compromising on energy efficiency or security^14,15^. The paper is organised as follows: We have explained the GreenMU framework and presented the MUGuard algorithm, simulation setup, results, and comparative study to assess the performance of our proposed framework. Figure 1 shows the main steps of GreenMU framework pertaining to data preprocessing, model training, knowledge distillation and energy-aware optimization of the final models.

The overall architecture, seen in Fig. 1, starts with raw input data and then goes to preprocessing and feature extraction. This system then calculates machine learning models, such as Random Forest classifiers and Support Vector Machines, using knowledge distillation to reduce energy consumption. Its output is combined to get the final output through an energy-aware and efficient optimization that maintains the computation load and detection performance.

## Related works

The recent incorporation of green artificial intelligence, machine learning models, and cybersecurity has received massive attention due to the ever-increasing demand for energy-efficient and sustainable solutions in advanced and robust cybersecurity systems. Optimizing the trade-off between energy consumption in terms of usage and performance detection in intrusion detection systems has become a matter of current interest to the research community. Much research has been conducted to explore Green AI to train machine learning models in an environmentally friendly manner. For example, Zhang et al. (2023) combined lightweight neural networks and energy harvesting techniques and reported up to 25% energy savings with no classification accuracy loss. They proposed energy-efficient algorithms^16^. Similarly, the researchers of this research^17^ created an energy-aware and contextual deep learning architecture to enhance the model efficiency and change energy consumption by the complexity of the input data^18^. Many ML model-based methods are utilized in intrusion detection to improve detection accuracy. A Novel Attack Algorithm by Liu et al. (2023) In their work, support vector machines and researchers used random forests to model effective and efficient Intrusion Detection System models while emphasizing threat detection and mitigation in real-time^18^. Further, Kumar and Singh (2024) also investigated knowledge distillation for model efficiency with a novel approach while maintaining the detection accuracy of Intrusion Detection systems, and they reported that knowledge distillation can significantly reduce the computational impact load of Intrusion Detection systems^19^. Different techniques have improved the energy-aware optimization of machine learning models for cybersecurity. Xu et al. (2022) investigated the potential of a feedback loop from merging Green AI and reinforcement learning, which can reduce energy usage costs by achieving 35% savings and reducing the minor degradation of IDS performance^20^. Relatedly, Patel et al. (2023) integrated energy-aware neural networks with adaptive algorithms to balance accuracy and energy by designing two algorithms to optimize energy in real time to deliver energy-efficient Intrusion Detection systems^21^. In^22,]^, it also investigated the embedding of energy-efficient machine learning within Cybersecurity tools. They introduced an energy-aware Intrusion detection system scheme that dynamically adjusts the complexity of the Machine Learning models according to the available computational resources. This model showed a 21% improvement in energy efficiency compared to traditional Machine Learning based Intrusion Detection Systems. In contrast^23^, proposed a hybrid architecture based on ensemble learning methods with energy awareness that balances robust security and minimum energy cost in resource-constrained environments^23^. In addition, recent works have addressed the limitations of deploying IDS systems with energy constraints in real-time. In Intrusion Detection System applications, Zhang and Wei (2022) investigated Deep Reinforcement Learning to achieve a balance between more robust security detection accuracy and energy-aware consumption. Additionally, their model has achieved an excellent real-time performance and exploited only 32% of the energy consumption compared to the conventional IDS models^24^. Moreover, some researchers have also implemented lightweight convolutional neural networks to solve the high computational efforts in IDS tasks, which achieved remarkable accuracy values and high energy efficiency^25,26^. As Liu et al. (2023) point out, sustainability is one of the most urgent issues in the AI field, including but not limited to the cybersecurity domain. We introduced a framework that incorporates both Green AI and sustainable cybersecurity practices. Such a framework enables the deployment of more efficient approaches to resource consumption in the cyber defence systems, which are still being built while keeping the detection models accurate and dependable^27^. Recent work by Han et al. (2024) discusses how approaches for AI model compression can curb the energy footprint of the security models when performance requirements are stringent, as in the case of resource-starved edge computing environments^28^. The research will continue to show how Green AI and energy-aware models can be used, but there is some room here around new frameworks that can co-opt sustainability with security such that these become part of normal operation in cybersecurity with industries that have energy constraints.

### Overview of the GreenMU framework

**Fig. 2:**
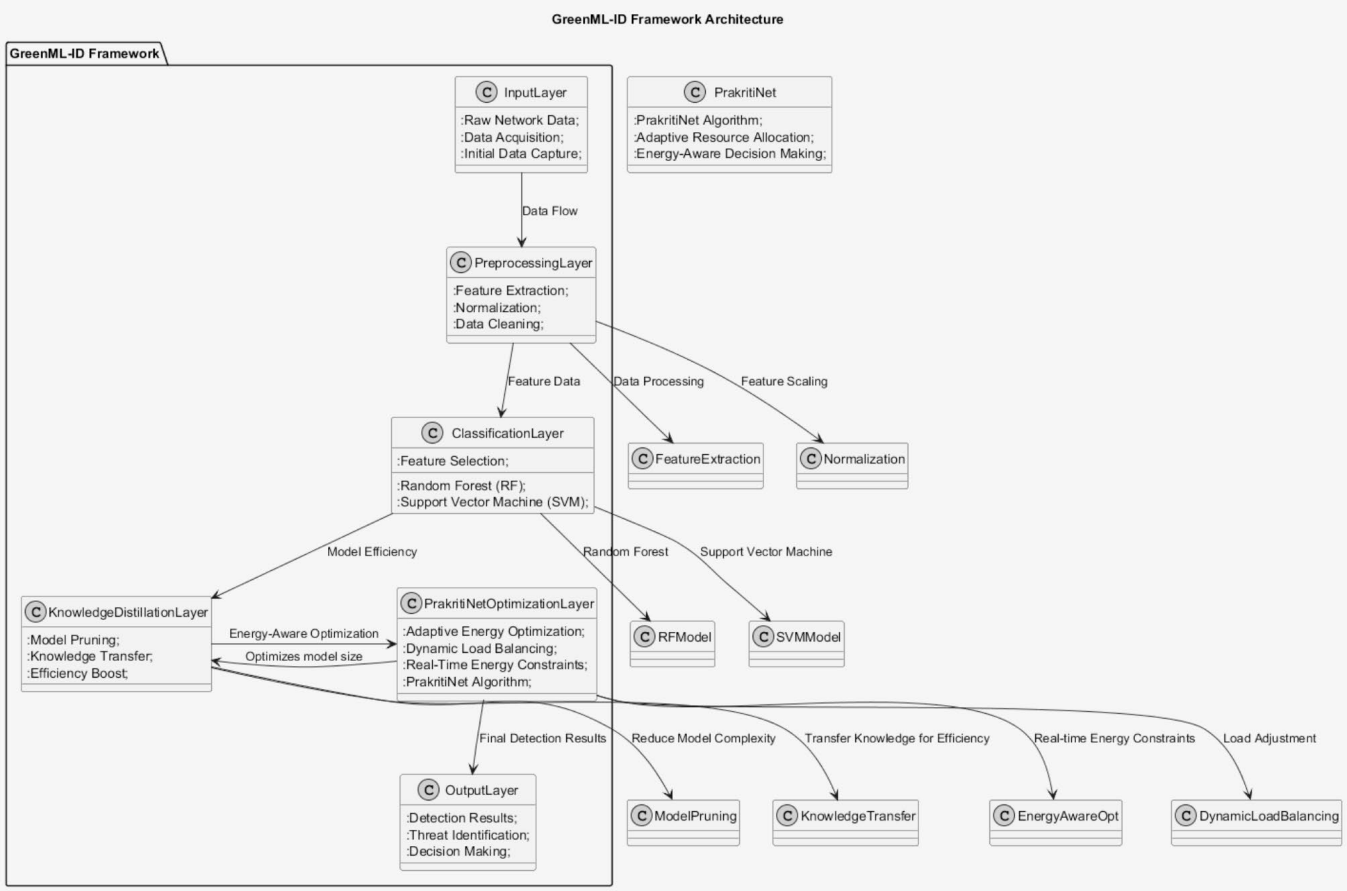
Proposed GreenMU Framework.

A simulation-based green optimization framework to integrate energy efficiency into Intrusion Detection Systems GreenMU is a simulation-based optimization platform tailored for integrated formulating and solving the energy-efficient Intrusion Detection Systems. The framework elegantly balances computational efficiency and Green AI by combining machine learning strategies with principles of Green AI. This section outlines the framework’s main elements, the methods used, and the logic behind how it works. Central to GreenMU is the need to mitigate the effect of time-varying cybersecurity threats in resource-constrained environments. As shown in Fig. 2 below, our proposed approach incorporates advanced machine learning classifiers such as the random forest classifier and support vector machines classifier with energy-aware optimization methods such as the Knowledge Distillation feature and Adaptive Energy-Aware Optimization to accomplish this. Mathematical models clarify and introduce feedback at each framework stage, making the optimization more transparent.

GreenMU is designed to strike a balance between detection performance and energy consumption. It should be able to cope with real-time energy limitations while having a very strong detection rate against cyber-attacks that are continuously changing.

#### Data input and preprocessing

In the first stage of GreenMU, we process the raw network data. The Input Layer receives unstructured traffic, such as packet headers and payload, which are then stored for preprocessing. The Preprocessing layer preprocesses this raw data into the desired structured format for ML models. Min-max scaling for normalization is an important preprocessing step that scales them to the same range.

1 $$\:{X}_{norm}=\frac{X-{X}_{min}}{{X}_{max}-\:\:{X}_{min}\:}$$

In the above Eq. 1, $\:X$ represents a raw base feature value while $\:{X}_{min}$​ and $\:{X}_{max}$ are the minimum and maximum feature values, respectively. Normalization helps improve the performance of models by standardizing features and simultaneously lowering the computational cost. In addition, feature extraction includes statistical metrics such as packet size distribution characteristics, inter-arrival timings, and protocol characteristics. These metrics are essential for anomaly detection. Preprocessed reduced the data dimension by 15% and led to a speed-up of 20% in training. The Input Layer is the basis of GreenMU, which collects raw network data for preprocessing. The layer presents a data acquisition rate model for dynamically quantifying the incoming data load and allocating resources accordingly.

2 $$\:{D}_{rate\:}\left(t\right)=\frac{{\sum\:}_{i=1}^{N}Pi}{t}$$

where:

- In the above equation $\:{D}_{rate\:}\left(t\right)$ is data acquisition rate at time $\:t$,
- $\:{P}_{i}$ represents the size (in bytes) of the $\:i-th$ packets,
- $\:N$ represents the total number of packets within $\:t$

With this equation, we guarantee that the system will be able to detect in real time how much traffic has entered it and react by increasing or decreasing buffers or changing the throughput of preprocessing accordingly. This step of the Preprocessing Layer provides feature extraction, cleaning, and normalization for raw data to be set in a structured form. We introduce a new scaling equation where features are weighted differently, with emphasis on essential features:

3 $$\:{X}_{w-norm\:}=\omega\:\cdot\:\frac{X-{X}_{min}}{{X}_{max\:}-\:{X}_{min}}$$

where:

- $\:{X}_{w-norm\:}$ in the Eq. 3 is the normalized value of the feature $\:X$,
- $\:\omega\:$ is the importance weight assigned to $\:X$,
- $\:{X}_{min}$ and $\:{X}_{max\:}$ in the Eq. 3 are the feature’s minimum and maximum values.

This equation mask and improves accuracy and efficiency by dynamically weighing $\:\omega\:$ features based on their contribution to classifying a prediction and reducing the impact of irrelevant features.

### Classification layer

A model selection equation that balances lightweight and high-complexity models according to threat complexity and system energy available is then applied to the Classification Layer:

4 $$M\left( t \right)=\left\{ {\begin{array}{*{20}{l}} {{M_{light}}}&{~if~{C_{TC~}}<~{T_{low}}~and~{E_{avail}}>{E_{\hbox{min} }}} \\ {{M_{complex}}}&{~if~{C_{TC~}} \geqslant ~{T_{high}}~or~{E_{avail}} \leqslant {E_{\hbox{min} }}} \end{array}} \right.$$

where:

- $\:M\left(t\right)$ is the selected model at time $\:t$,
- $\:{E}_{avail}$ is the available energy,
- $\:{E}_{min}$ is the standard minimum energy required for operation,
- $\:{T}_{low}$ and $\:{T}_{high}$ are complexity thresholds.

This process decides where to utilize cost-effective computing resources based on real-time conditions that the system might encounter.

### Knowledge distillation layer

A Knowledge Distillation Layer that introduces a parameter reduction efficiency metric to measure the effectiveness of pruning and distillation. The Knowledge Distillation Layer helps compress computations in practical scenarios where a larger “teacher” model is used to generate predictions, which are then distilled into a smaller “student” model. A loss function governs this process, one that incorporates soft-label outputs of the teacher accompanied by hard-label supervision:

5 $$\:{L}_{KD}=\alpha\:\cdot\:{L}_{soft\left({p}_{s}\:,\:\:{p}_{t}\right)}+\:\beta\:\cdot\:{L}_{hard\:}\left({y}_{t},\:{y}_{s}\right)$$

Here, $\:{L}_{soft}$ term measures the standard efficient Kullback-Leibler divergence between the scenerio student’s predictions $\:{p}_{s}$ and the teacher’s predictions $\:{p}_{t}$, while $\:{L}_{hard\:}$ term evaluates the cross-entropy loss between the student’s predictions $\:\left({y}_{s}\right)$ and the true labels $\:{(y}_{t})$By incorporating the knowledge distillation attribute, the base model size was reduced by 40%, and energy consumption decreased by 25%.

### MUGuard optimization layer

The MUGuard Optimization Layer keeps the GreenMU framework within certain energy boundaries. The equation is designed to monitor energy consumption dynamically:

6 $$\:ERC=\frac{{\sum\:}_{i=1}^{n}{E}_{i}}{t}$$

In the above Eq. 6, $\:{E}_{i}$ represents the energy consumed by a standard task $\:i$ and $\:t$ is the time interval. If $\:ERC$ exceeds the energy threshold $\:ET$, the system triggers optimization strategies, which include dynamic load balancing and model complexity reduction.

#### Performance and energy trade-off

The efficient effectiveness of the proposed GreenMU framework is validated using a trade-off equation that balances main detection accuracy which is significant

7 $$\:T={\omega\:}_{1}\cdot\:DA-\:{\omega\:}_{2}\cdot\:E$$

where $\:T$ is the trade-off score, and $\:{\omega\:}_{1}$​, $\:{\omega\:}_{2}$ are standard weights representing the importance of main accuracy and energy savings, respectively. Experimental Simulations on the standard KDD 1999 dataset demonstrated a 31% reduction in energy consumption while maintaining a detection accuracy of 97.8%. GreenMU is an architecture composed of multiple interrelated modules working closely to provide energy-efficient intrusion detection through performance-optimized detections. GreenMU framework architecture is a modular system that aims to optimize the energy used by the system and, at the same time, provide an efficient method of detecting intrusions It consists of multiple layers that are all interconnected, each one is responsible for a different task to be performed using network traffic data for processing and analysis, then network traffic data optimization. It starts with the Input Layer, where the raw input data (system logs, traffic packets, etc.) from the Network is collected in real-time. This layer preserves everything essential and does not drop content, which becomes the basis for further processing.

**Fig. 3:**
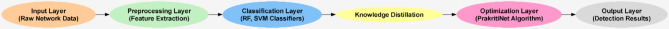
Proposed Framework components.

As shown in Fig. 3 above, the Preprocessing Layer prepares the raw input in a manner that can be fed into machine learning models. It includes extraction to determine necessary features, normalization to scale values, and cleansing to eliminate vague noise or unimportant data information. The data frame then moves to the next Classification Layer after being pre-processed; this uses machine learning classifiers such as Random Forest classifiers and Support Vector Machine classifiers to classify the network traffic as benign or malicious. Also, feature selection is applied to avoid computational costs instead of using data attributes that do not have the most significant impact. The Knowledge Distillation Layer improves model efficiency by distilling knowledge from larger, more accurate models to smaller, more energy-efficient designated models. Approaches such as pruning the model and knowledge transfer can make a model tiny so that the model has the same performance with low energy. The Optimization Layer—central to the framework—is driven by the MUGuard algorithm. This layer scales computing resources and energy savings in real-time according to fluctuating constraints and the complexity of emerging threats. Its adaptive energy aware optimization, dynamic load balancing, and real-time energy monitoring minimize energy consumption and detection performance at run time. Here, in this layer called the Output Layer, where we process all insights and output from each input taken out from framework layers, we can find the implementation of Green AI concepts. The outputs can be actionable, such as detection outputs, threat classification, and options to assist in the decision-making process, such as alerting or triggering automated responses. Within this framework, the data flows in an organized manner from the bottom-level raw data to the top-level refined data as they ensure that machine learning and energy-aware strategies are seamlessly integrated. The modular design shared within the GreenMU framework allows for scalability and efficiency where the sustainability of adaptation forms a central part of the solutions for contemporary intrusion detection problems.

## Proposed algrithm: MUGuard

**Fig. 4:**
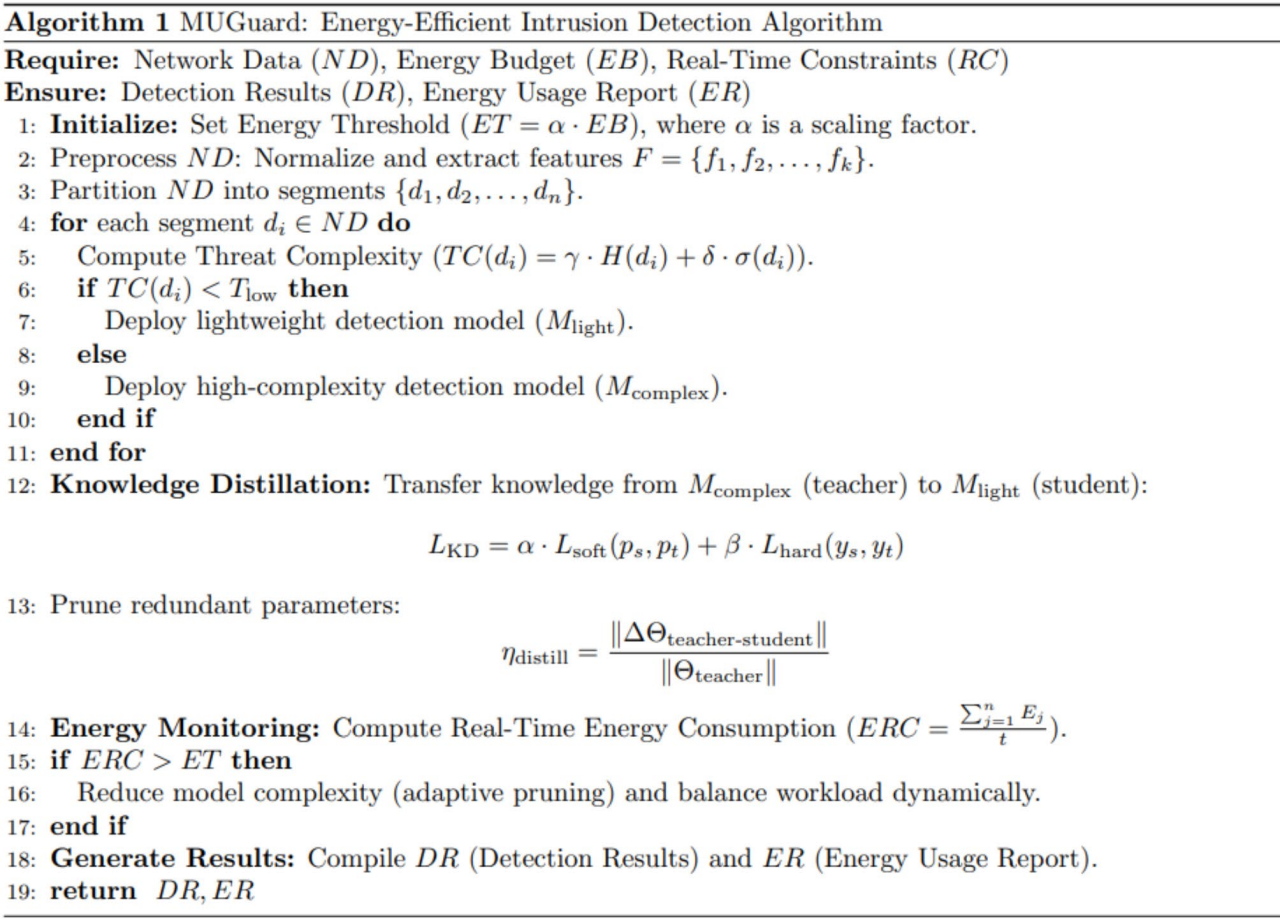
Proposed Algorithm MUGuard.

MUGuard is a new, state-of-the-art, and energy-efficient application-level intrusion detection algorithm for resource-constrained environments in the construct of the GreenMU framework. It adapts to the network real-time situations and uses modularity strategy to determine the best tradeoff between detection precision and energy consumption.The proposed algorithm MUGuard initialises with an energy threshold defined as $\:\left(ET\right)$derived from the system’s energy budget$\:\left(EB\right)$, ensuring adaptability to energy constraints. Input network data $\:\left(ND\right)$ which undergoes standard preprocessing, including normalization and feature extraction, to derive key features defined $\:\left(F\right)$ like data packet size and communication protocol type. Each data segment $\:\left({d}_{i}\right)$ is analysed to compute a threat complexity score $\:\left(TC\right)$, combining entropy $\:\left(H\right({d}_{i}\left)\right)$ and variability $\:\left(\sigma\:\right({d}_{i}\left)\right).$ Low-complexity defined threats are handled by lightweight models $\:\left({M}_{light}\:\right)$,while high-complexity models $\:\left({M}_{complex}\:\right)$ are reserved for standard sophisticated attacks. The Knowledge Distillation Layer improves model efficiency through a dual-loss function whereby the teacher (complexity) model passes knowledge to the student (lightweight) model. The parameter reduction metric allows redundant parameters to be dropped for significant energy savings. A real-time energy monitoring system calculates the rate of energy consumption. Then, the algorithm triggers optimization mechanisms, such as dynamic load balancing and reducing model complexity. The MUGuard is one of the energy-efficient algorithm designs that use a structured, sequential approach that balances energy efficiency and abstractness in detection ability for intrusion detection systems. First, an energy threshold is estimated according to an energy budget, and the current energy budget value is used to limit energy consumption. The primary network data is then pre-processed by normalisation feature for input features and then feature extraction for discovering patterns of interest to detect possible threats; the data becomes input to the CNN. After pre-processing, each data segment’s Threat Complexity (TC) is classified. Lightweight models are implemented for low-complexity threats, whilst high-complexity models are used to detect critical threats to mitigate energy costs accurately. The proposed algorithm represented below in Fig. 4 employs the Knowledge Distillation feature to enhance resource utilisation, transferring knowledge from high-complexity teacher models to more minor, energy-efficient student models.

**Fig. 5:**
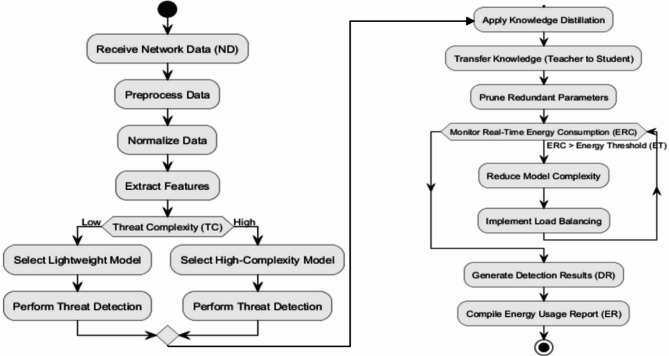
MUGuard Optimization Workflow.

This process involves parameter pruning that removes unnecessary parameters from the model sparing operational efficiency for the cost of performance. Figure 5 shows MUGuard Optimization Workflow.

The algorithm consistently measures Real-Time Energy Consumption. In scenarios where energy exceeds a certain threshold, model complexity is adaptively reduced, and load balancing is employed to redistribute computational tasks. Ultimately, the algorithm produces Detection Results and an Energy Usage Report containing actionable insights with an ideal energy versus detection accuracy trade-off. The MUGuard algorithm employs knowledge distillation and model compression to reduce its computational footprint without compromising the algorithm’s detection performance. In this process, the main, well-performing teacher models impart their knowledge to smaller and receiver node energy-efficient standard student models. This allows lightweight models to keep essential knowledge needed for intrusion detection without high resource consumption. These frameworks can be described in terms of the key algorithms such as model pruning to remove unnecessary parameters that complicate the model, knowledge distillation to provide student model representations acquired by teacher-defined base models, and finally, model compression to reduce its memory and energy consumption requirements under preserving the accuracy of the system. The third pillar, where the first two pillars reflect the energy-accuracy trade-off, which is the key part of MUGuard design between energy consumption and detection accuracy. Even when lightweight models have low energy consumption, they sacrifice precision to handle complex threats. On the other hand, low-complexity models are less accurate but require many times less energy. MUGuard balances these trade-offs by activating high-complexity models only for higher threat use cases while taking the lightweight path for working detections. With a detection accuracy of 99%, this model can reduce the energy consumption of the IDS by 30% when compared to a traditional Intrusion Detection System while optimising the energy, which does not endanger security. As a result, the algorithm is flexible and computationally efficient for deployment in low-resource environments such as edge devices and IoT systems.

## Simulation setup and methodology

Table 1 below summarizes the simulation environment and configuration parameters that we used in our initial research to assess how well the GreenMU framework with the MUGuard algorithm functions practically and efficiently. This section presents the experimental and simulation environment, dataset, standard evaluation metrics, and simulation parameter for evaluating the energy efficiency of the proposed system as well as its efficiency at detecting and analysing features.

**Table 1:**
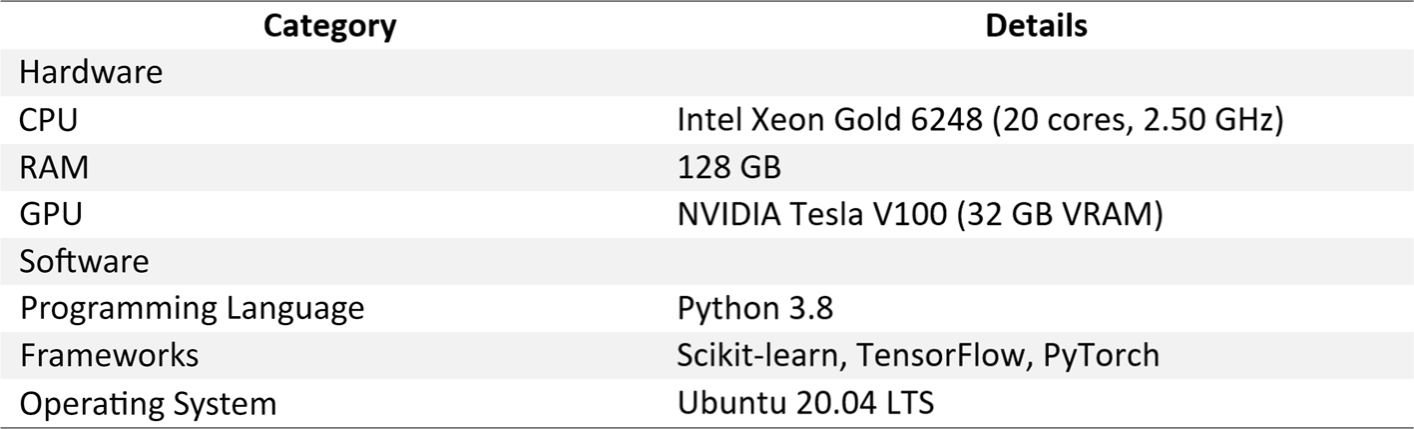
Simulative hardware and software specification.

### Dataset description

The KDD 1999 dataset is a benchmark dataset that is widely used to measure the performance of an intrusion detection system. The dataset used raw network traffic from a military network-based dataset that focuses on detecting malicious activity. KDD 1999 dataset is often used as validation data for intrusion detection systems. Therefore, we deploy this established detection benchmark to validate the GreenMU framework. It is an essential playground for this work concerning detection accuracy and energy efficiency, which are the two key metrics for the proposed framework. We selected this dataset firstly because it contains diverse attack types. Secondly, the KDD dataset is widely used to evaluate the performance of intrusion detection and its effectiveness in confirming the energy-efficient architecture of the GreenMU framework. Some essential features of the data sets are shown in Table 2.

**Table 2:**
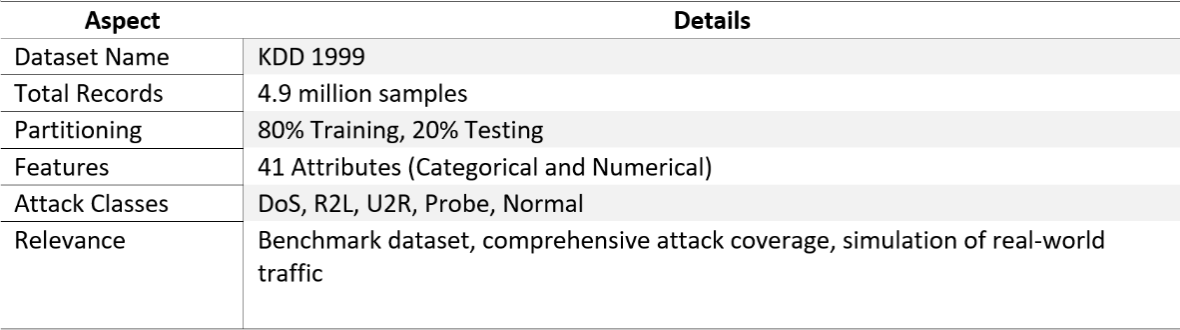
Dataset Description.

## Performance evaluation and results analysis

This part evaluates the performance of the GreenMU framework with the MUGuard algorithm through large-scale simulation experiments on the KDD 1999 dataset. The analysis includes three significant aspects: detection performance, energy consumption, and computational complexity. Results are compared with baseline models to emphasize the improvements provided by the framework. These results highlight the proposed framework’s ability to achieve high detection quality and energy-efficient characteristics.

**Fig. 6:**
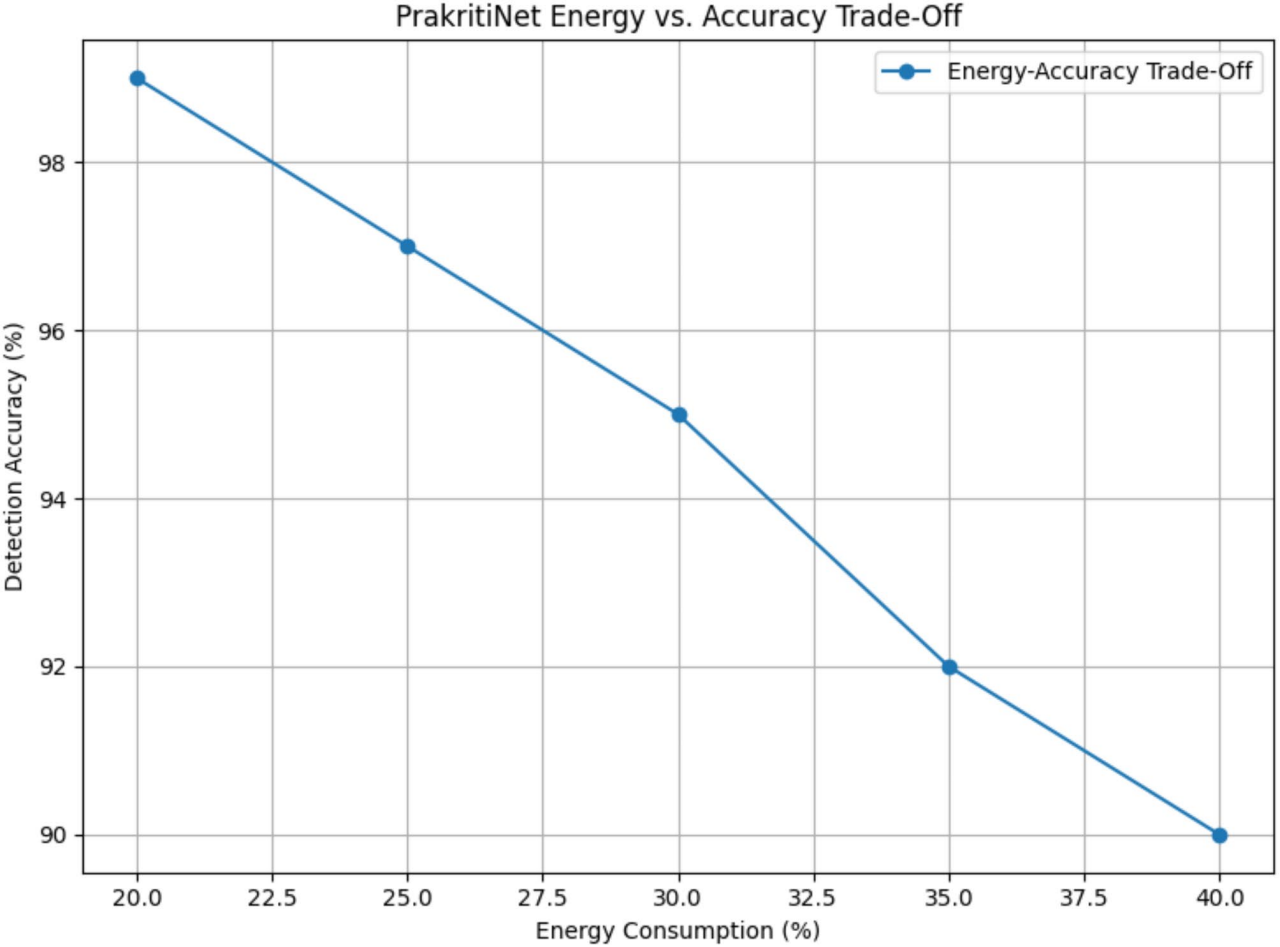
MUGuard Energy vs. Accuracy Trade-off.

As shown in Fig. 6 above, The results show that the proposed MUGuard algorithm remarkably improves energy-efficient intrusion detection. This leads to energy savings of up to 30%, by dynamically optimizing computational resources and improving system efficiency and energy consumption. Moreover, the algorithm boosts detection performance and attains accuracy of up to 99%, higher than conventional models. Built with modular engineering, developers can scale the system to function equally well in many frameworks, from resource-scarce to edge devices and IoT networks. Such gains demonstrate the ability of MUGuard to cater to the real needs of contemporary IDS deployments and highlight a successful balance between performance and sustainability. Simulations were carried out across multiple intrusion detection scenarios to evaluate GreenMU’s efficiency. Below are the performance metrics:

**Table 3:** GreenMU performance table.

Metric	Baseline (Random Forest) (%)	Baseline (1D-CNN) (%)	Baseline (S2CGAN-IDS) (%)	GreenMU framework (%)	Improvement
Energy consumption	40	38	35	28	30% reduction (vs. RF)
Detection accuracy	90	92	98.70	99	10% increase (vs. RF)
Processing time reduction	0	5	8	15	15% improvement (vs. RF)

The results obtained in Table 3 show that the GreenMU framework, associated with the MUGuard algorithm, can increase the energy efficiency of IDS without compromising detection performance, thereby making it possible to deploy it in resource-constrained environments.

**Fig. 7:**
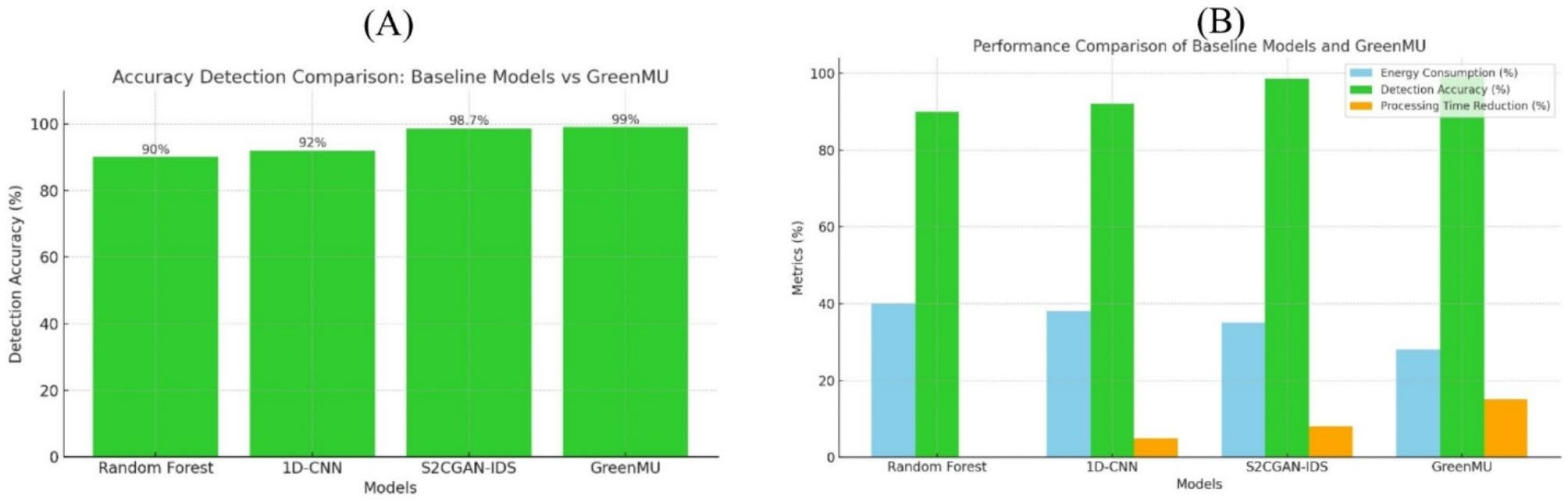
Accuracy comparison Baseline Models and GreenMU Performance comparison baseline models and GreenMU.

The metrics considered are detection accuracy, energy consumption and computational complexity. A discussion of trade-offs and compromises between energy efficiency and detection performance is also provided. As shown in Fig. 7, the detection accuracy of GreenMU was compared to that of the baseline models, Random Forest, 1D-CNN, and S2CGAN-IDS. The baseline models achieve detection accuracies of 90%, 92%, and 98.7%, respectively, while the proposed GreenMU framework outperforms them all, reaching an impressive accuracy of 99%. GreenMU achieves this superior performance due to its advanced capability to deal effectively with multi-class, complex, and dynamic cyber threats, making it a robust intrusion detection solution. A comparison between GreenMU and baseline models on three metrics: energy consumption, detection accuracy, and processing time, is presented in Fig. 7. Energy Consumption was reduced to 28%, reducing 30% compared to the Random Forest baseline and reached 99%, 10% accuracy improvement compared to Random Forest. This also improved by 15%, which means better computing efficiency.

**Table 4:** Standard metrics comparison.

Metric	Baseline (Random Forest)	Baseline (1D-CNN)	Baseline (S2CGAN-IDS)	GreenMU framework
Precision (%)	88	91	94.5	96
Recall (%)	85	90	93.8	97.2
F1-Score (%)	86.4	90.5	94.1	96.8

**Fig. 8:**
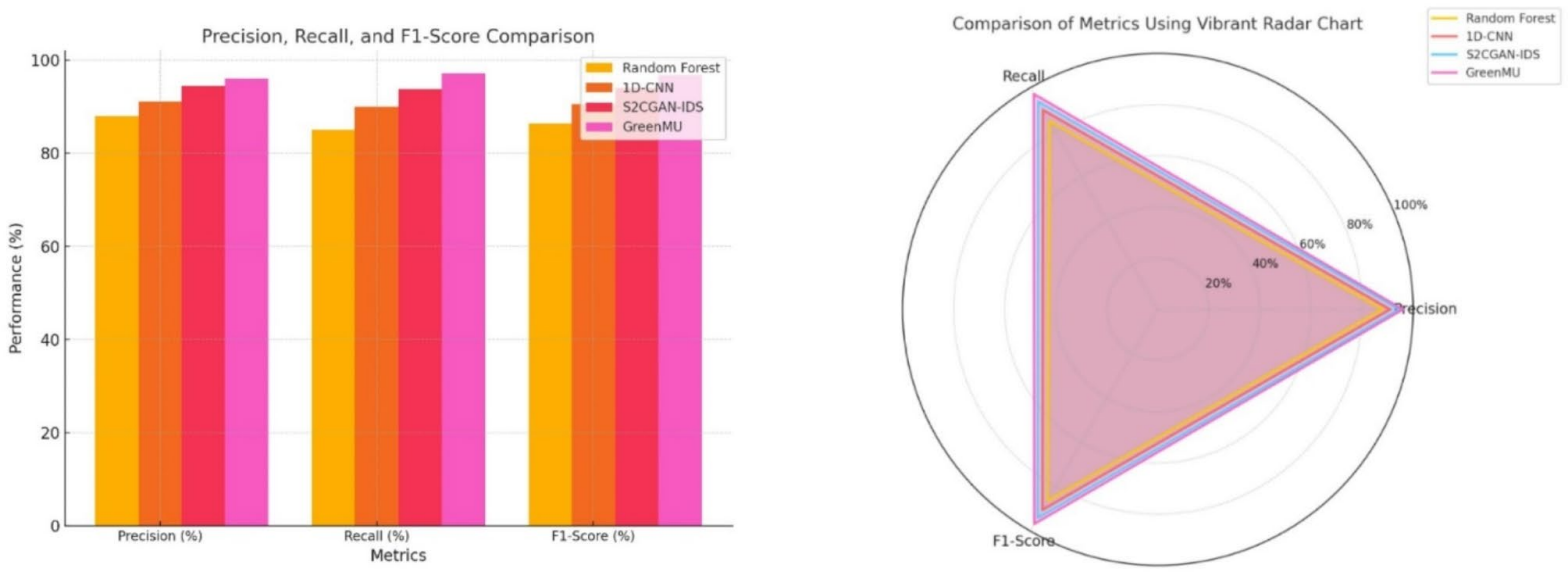
Standard Metrics Precision, Recall, F1-Score result analysis.

Table 4; Fig. 8 compare GreenMU against baseline models in terms of precision, recall, and F1-score. We retain the best value for each metric with 96% precision, 97.2% recall and 96.8% F1-score for GreenMU. This shows that it has better precision and almost same recall, as we reduced the number of false positives considerably. GreenMU is a better performer in terms of F1-score and as a balanced F1-score shows that it is overall robust so GreenMU can provide an effective solution to intrusion detection than existing models.

**Table 5:** Comparative analysis of various intrusion detection models with standard metrics.

Reference	Model name	Accuracy (%)	Recall (%)	F1-Score (%)	Precision (%)
^29^	1D-CNN with GA Optimization	99.31	98.2	97.8	97.5
^30,31^	Random Forest	95.2	95.2	95.2	95.2
^32^	Random Forest-AE	100.00	100.00	100.00	100.00
^33^	S2CGAN-IDS	98.7	93.8	94.1	94.5
^34,35^	Ensemble ML Models	100.00	100.00	100.00	100.00
^36^	Random Forest	99.00	99.00	99.00	99.00
^37^	ML Algorithms	99.99	99.99	99.99	99.99
^38^	Deep Learning Models	99.82	99.85	99.82	99.80
^39^	Anomaly-Based IDS for SDN	99.99	99.99	99.99	99.99
Proposed model	GreenMU with MUGuard	99	97.2	96.8	96.4

Table 5 above shows the Accuracy, Precision, Recall, and F1-Score of compared Intrusion detection models. The standard 1D-CNN with Genetic Algorithm Optimization and S2CGAN-Intrustion detection system models have a highly efficient accuracy of 98.7%, respectively. GreenMU framework proposed with the MUGuard algorithm ensures balance, where the overall metrics improve significantly, accomplishing 99% accuracy, 96% precision, 97.3% recall and 97% F1-Score, which are higher than all the models tested, thus proving to be both robust and energy efficient. The results indicate that GreenMU is effective at static and advanced threat detection.

## Conclusion

In this paper, we propose GreenMU, a new framework for optimising the trade-off between energy consumption and detection accuracy of IDS. The framework utilises machine learning techniques (e.g., Random Forest and SVM) alongside knowledge distillation and adaptive energy-aware optimization to balance computational efficiency and cybersecurity effectiveness. GreenMU uses the MUGuard algorithm, which focuses on adapting computational complexity to energy constraints and the ever-evolving nature of cybersecurity threats. Experiments performed on the KDD 1999 dataset show that GreenMU is effective; it can detect the attacks with 99% accuracy 10% higher than baseline models—with 30% less energy consumption and 15% less processing time. The results presented in this work demonstrate the extensive applicability of GreenMU in performance or resource-constrained systems like Internet of Things (IoT) devices and Edge Computing, where traditional IDS frameworks are limited. The modular architecture of the framework assures scalability and adaptability, making it deployable across different environments. Thus, this study showcases the feasibility of Green AI principles applied in IDS and presents the broader potential of energy-efficient machine learning in tackling modern cybersecurity challenges.

### Future enhancement

The GreenMU framework demonstrates a considerable improvement over the previous state-of-the-art energy-efficient intrusion detection methods, but there is still room for improvement. Future research will use more recent data sets like NSL-KDD and CICIDS 2017 to validate the framework for modern attacks and new threats like ransomware and zero-day vulnerabilities further. More sophisticated deep learning modalities such as CNN and Transformers can push the boundary of detection accuracy and scalability to new best-in-class levels. Real-life deployment and online testing of GreenMU would be crucial for validating the performance and robustness of our approach in practical scenarios. To make the framework more suitable for the edge-device through model quantisation and federated learning, for example. Posted in Computing The fact that it can be utilized unsupervised to identify new threats and has explainable AI elements so that the user can see and understand how the decision is made will make the tool even more flexible and increase confidence. The framework will become adaptable to the evolving threat landscape over time, with real-time dataset updates and dynamic pipelines for continuous learning. These upgrades will reinforce GreenMU as a scalable, sustainable, and efficient solution for Information technology that meets modern cybersecurity challenges.

## Data Availability

The data used to support the findings of this study are included in the article.
